# Evaluation of human-papillomavirus testing and visual inspection for cervical cancer screening in Rwanda

**DOI:** 10.1186/s12905-018-0549-5

**Published:** 2018-04-24

**Authors:** M. Chantal Umulisa, Silvia Franceschi, Iacopo Baussano, Vanessa Tenet, Mathilde Uwimbabazi, Belson Rugwizangoga, Daniëlle A. M. Heideman, Anne M. Uyterlinde, Teresa M. Darragh, Peter J. F. Snijders, Felix Sayinzoga, Gary M. Clifford

**Affiliations:** 1 Ministry of Health of Rwanda, Kigali, Rwanda; 2 Cancer Epidemiology Unit, Aviano National Cancer Institute IRCCS, Aviano, Italy; 3 International Agency for Research on Cancer, 150 cours Albert Thomas, 69372 Lyon Cedex 08, France; 4 Muhima District Hospital, Kigali, Rwanda; 5 Department of Pathology, University Teaching Hospital of Kigali, Kigali, Rwanda; 6 University of Rwanda School of Medicine and Pharmacy, Kigali, Rwanda; 7 Department of Pathology, VU University Medical Center, Amsterdam, the Netherlands; 8 University of California, San Francisco, CA USA

**Keywords:** Human papillomavirus, Visual inspection, Cervical cancer, Screening, Rwanda

## Abstract

**Background:**

A pilot screening campaign in Rwanda, based on careHPV-testing followed by visual inspection with acetic acid triage (careHPV+VIA triage), was evaluated against other WHO-recommended screening options, namely HPV screen-and-treat and VIA screen-and-treat.

**Methods:**

764 women aged 30-69 underwent at visit 1: i) VIA, and cervical cell collection for ii) careHPV in Rwanda, and iii) liquid-based cytology and GP5+/6+ HR-HPV PCR in The Netherlands. All 177 women positive by VIA, careHPV and/or PCR were recalled, of whom 84% attended. At visit 2, VIA was again used to triage screen-positive women for treatment and to obtain biopsies from all women either from visible lesions or at 12 o’clock of the squamocolumnar junction. Cross-sectional screening indices were estimated primarily against histological high-grade squamous intraepithelial lesions or worse (hHSIL+), after imputation of missing histology data, based on 1-visit or 2-visit approaches.

**Results:**

In a 1-visit screen-and-treat approach, VIA had sensitivity and specificity of 41% and 96%, respectively, versus 71% and 88% for careHPV, and 88% and 86% for PCR. In a 2-visit approach (in which hHSIL+ imputed among women without visit 2 were considered untreated) careHPV sensitivity dropped to 59% due to loss of 13% of hHSIL+. For careHPV+VIA triage, sensitivity dropped further to 35%, as another 24% of hHSIL+ were triaged to no treatment.

**Conclusions:**

CareHPV was not as sensitive as gold-standard PCR, but detected considerably more hHSIL+ than VIA. However, due to careHPV-positive hHSIL+ women being lost to follow-up and/or triaged to no treatment, 2-visit careHPV+VIA triage did not perform better than VIA screen-and-treat.

**Electronic supplementary material:**

The online version of this article (10.1186/s12905-018-0549-5) contains supplementary material, which is available to authorized users.

## Background

Cervical cancer is the most commonly occurring female cancer in Rwanda, with a high incidence rate typical of many sub-Saharan African countries (42 cases per 100,000 women per year [1]). In response to this burden, Rwanda embarked upon a national plan for cervical cancer screening [2] and, in 2011, was the first African country to initiate a national vaccination program against human papillomavirus (HPV) [2, 3], the necessary cause of cervical cancer [4].

In order to also reduce cervical cancer among older cohorts of women unprotected by the vaccination program, in 2013, the Rwandan Ministry of Health (MoH) initiated a screening campaign. This campaign was based on primary careHPV testing and the use of visual inspection with acetic acid (VIA) to triage careHPV-positive women for treatment [2]. Indeed, WHO guidelines for cervical screening in low and middle income countries (LMIC) include an algorithm of high-risk (HR) HPV-testing followed by VIA triage as one of its three recommended screen-and-treat options [5]. Although some experiences have since been reported [6–8], WHO recommendations acknowledged that there was no evidence on the effectiveness of VIA triage among women known to be HR HPV-positive. The prediction of the efficacy of a HPV + VIA triage approach, as well as its recommendation over a VIA screen-and-treat approach, came from combining evidence of separate performances of HPV and VIA [5].

We nested an evaluation of the 2013 Rwandan MoH screening campaign within the framework of a population-based HPV survey performed in collaboration between the Rwandan MoH and the International Agency for Research on Cancer (IARC) to jointly monitor the impact of HPV vaccination [9] and cervical screening. The careHPV-testing followed by VIA triage algorithm was principally evaluated against the gold standard of high-grade squamous intraepithelial lesions or worse (HSIL+) as detected by histology, and was compared with the other two WHO recommended screen-and-treat options for LMICs, namely HPV screen-and-treat and VIA screen-and-treat.

## Methods

### Population

Between July 2013 and May 2014, during a population-based survey of HPV prevalence, 2508 women aged 18-69 years were invited and underwent collection of cervical cells in Muhima hospital or eight other health centers in Nyarugenge district, Kigali, Rwanda. Study procedures have been described in detail elsewhere [9]. In short, exfoliated cervical cells were obtained from all women using a cytobrush (Rovers Medical Devices, The Netherlands), and were placed in PreservCyt medium (Cytyc-Hologic, Marlborough, MA, USA) for the performance of liquid-based cytology and HPV detection using GP5+/6+−based polymerase chain reaction (PCR) at VU University Medical Center, Amsterdam (see below), the results of which have already been published [9].

**Fig. 1:**
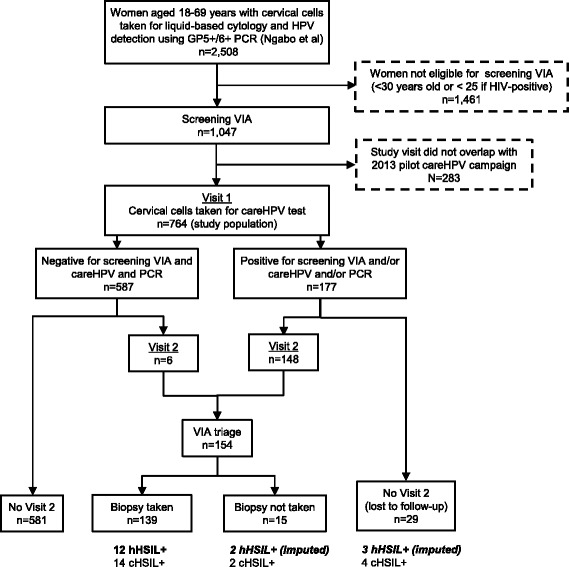
Flow chart of study population, procedures and outcomes, Rwanda 2013-14

Participants aged ≥ 30 years (or ≥ 25 years if known to be HIV-positive) (*n* = 1062) were also offered visual inspection at their first study visit. The cervix was visually inspected after application of dilute acetic acid, with results reported according to the IARC criteria [10]. This test is hereafter referred to as “screening VIA” (to differentiate it from VIA triage that was used at a second visit to decide treatment, see below). Furthermore, for a subset of these women for whom their study visit coincided with the implementation of the 2013 Rwandan MoH pilot careHPV screening campaign in the same hospital [2], a second cervical cell sample was collected with a careBrush and placed in a vial containing collection medium for careHPV testing. A total of 764 women with results for careHPV are the subject of the present report (Fig. 1).

The study had the approval of both the Research Ethical Board of the Rwanda Ministry of Health and the IARC Ethics Committee. Written informed consent was obtained from all study participants.

### CareHPV

Samples were tested using the careHPV platform (Qiagen Corporation, Gaithersburg, MD, USA) installed in Muhima Hospital, according to the manufacturer’s instructions. The careHPV test is a validated signal-amplification, rapid batch diagnostic test for the detection of DNA of 13 HR-HPV types (HPV16, 18, 31, 33, 35, 39, 45, 51, 52, 56, 58, 59, 68) and HPV66. CareHPV results were communicated as soon as possible to the screening clinic to arrange the follow-up of careHPV-positive women. However, due to the practical logistics, careHPV results were not available on the same day as the initial visit.

### Liquid-based cytology

Slides for liquid-based cytology were prepared from cell samples in PreservCyt medium using a Thin Prep 3000 processor (Cytyc-Hologic), stained according to manufacturer’s instructions and read at the Department of Pathology at the VU University Medical Center, Amsterdam, the Netherlands. Cytological diagnosis was made according to CISOE-A standards and was translated into the Bethesda 2001 terminology system [11].

### HPV testing by PCR

DNA was extracted from the PreservCyt sample using magnetic beads on a robotic system at the Department of Pathology at the VU University Medical Center, Amsterdam. β-globin PCR analysis was conducted to confirm the presence of human DNA in all specimens [12] and GP5+/6+−mediated PCR was used to amplify HPV DNA [13]. HPV positivity was assessed by hybridization of PCR products in an enzyme immunoassay with two oligoprobe cocktails that, together, detect 44 HPV types. HPV genotyping was subsequently conducted by reverse-line blot (RLB) hybridization of PCR products as described previously [14]. HR-HPV refers to 13 high-risk HPV types only (16, 18, 31, 33, 35, 39, 45, 51, 52, 56, 58, 59 and 68) [4]. Non-high-risk HPV types are ignored in the current analysis.

### Cervical disease assessment

One hundred and seventy seven women who were positive by screening VIA and/or by careHPV and/or for HR-HPV infection by PCR (for which results became available at a later date) were all recalled for a second visit, among whom 148 (84%) attended and 29 (16%) were lost to follow-up (Fig. 1). Six of 587 women negative for the three screening tests were also inadvertently recalled for a second visit.

At the second visit, VIA was again used to triage women for immediate treatment (by cryotherapy, thermocoagulation, or if ineligible, loop electrosurgical excision procedures) or not, according to the protocol of the Rwandan MoH careHPV screening campaign [2], hereafter referred to as “VIA triage”. In addition, for research purposes only, biopsies were to be taken from all women undergoing VIA triage, either VIA-directed to a visible lesion, or, in the absence of a visible lesion, randomly from 12 o’clock of the squamocolumnar junction, prior to any treatment. Histological confirmation of biopsies was performed at the Department of Pathology at the University Teaching Hospital of Kigali, and all biopsies from women with histological and/or cytological abnormalities were re-read by a specialist (TMD) reported according to LAST criteria [15] which became the reference diagnosis. Adequate histology results were obtained from 139 (90%) of 154 women who underwent VIA triage at the second visit, among whom 12 cases were diagnosed as hHSIL+ (including 1 invasive squamous cell carcinoma).

### Statistical analysis

Conventional screening indices of cross-sectional accuracy, including sensitivity, specificity, positive predictive value (PPV), negative predictive value (NPV), and their 95% confidence intervals, were calculated primarily against histological HSIL or worse (hHSIL+). Corrected indices were calculated after imputation of missing histology data. In the corrected model, pseudo-observations were created for women without a valid histology result, weighted by the probability of hHSIL+ among women with valid histology outcomes and the same combination of cytology, VIA, careHPV and PCR results [16–18]. Hence, in addition to the 12 confirmed hHSIL+, an additional 5 hHSIL+ were estimated among women without a valid histology outcome (Table 1). Only corrected indices are shown, but crude indices assuming that all women without valid histology had no hHSIL+ can also be calculated from the data described in Table 1. As secondary analyses, screening indices were also calculated against the outcome of cytological HSIL or worse (cHSIL+), or against a composite outcome of cytological and/or histological HSIL+ (composite HSIL+).

**Table 1:** hHSIL+ among 764 women with and without biopsies respectively, by combination of cytology, VIA, PCR and careHPV results

Cytology^1^	VIA^2^	PCR^3^	careHPV	*N*	Women with biopsy		Women without biopsy		All women	
					*N*	Confirmed	*N*	Estimated	hHSIL+	%
						hHSIL+		hHSIL+		
<cHSIL	–	–	–	587	6	0	581	0.0	0.0	0.0
	–	–	+	28	20	1	8	0.4	1.4	5.0
	–	+	–	42	28	1	14	0.5	1.5	3.6
	–	+	+	51	41	2	10	0.5	2.5	4.9
	+	–	–	27	24	0	3	0.0	0.0	0.0
	+	–	+	2	1	0	1	0.0	0.0	0.0
	+	+	–	3	2	1	1	0.5	1.5	50.0
	+	+	+	4	3	2	1	0.7	2.7	66.7
cHSIL+	–	+	–	3	2	2	1	} 1.3	} 4.3	}25.0
	–	+	+	14	10	1	4			
	+	+	+	3	2	2	1	1.0	3.0	100.0
				764	139	12	625	5	17	2.2

^1^ 19 missing cytology are considered cytology negative

^2^ Screening VIA at first visit

^3^ HR-HPV positivity for GP5+/6+-based PCR testing

*cHSIL* cytological high-grade squamous intraepithelial lesions, *hHSIL* histological high-grade squamous intraepithelial lesions, *HR-HPV* high-risk human papillomavirus, *PCR* polymerase chain reaction, VIA visual inspection with acetic acid

The performance of VIA, careHPV and PCR were first compared in a hypothetical scenario of single-visit screen-and-treat, treating all test-positive women at the same visit. In this model, the HSIL+ cases (imputed hHSIL+ and/or observed cHSIL+) among the 29 women without a second visit are assumed to be treated.

Secondly, based on the actual 2-visit approach that was used, we compared the Rwandan MoH careHPV screening campaign algorithm of careHPV primary testing at the first visit followed by VIA triage to decide treatment or not, against the counterfactual option of treating all careHPV-positive women at the second visit. In this analysis, the HSIL+ cases among the women without a second visit are lost to follow-up and remain untreated.

## Results

Of the 764 screened women, mean age was 43 years [interquartile range = 35-49 years], 84% were literate, 66% were currently married, 56% reported more than one lifetime sexual partner, 35% were known to be HIV-positive, and only 2.6% reported previous attendance to cervical screening (Additional file 1: Table S1).

Table 2 shows indices of cross-sectional accuracy by different primary screening methods, firstly in a 1-visit screen-and-treat scenario. Positivity rate was 5.1% for VIA screen-and-treat, 13.4% for careHPV and 15.7% for PCR. CareHPV showed higher sensitivity against hHSIL+ (71%) than VIA (41%), but lower than that of PCR (88%). NPV was also higher for careHPV (99.2%) than screening VIA (98.6%) but lower than that of PCR (99.7%). Specificity of careHPV (89%), on the other hand, was lower than for screening VIA (95%), and similar to PCR (87%). PPV for hHSIL+ was highest for screening VIA (17%).

**Table 2:** Screening algorithm accuracy to detect 17 hHSIL+ among 764 women aged 25-69 years, after correction for missing histology outcomes

	Women treated		hHSIL+ treated	Sensitivity (95%-CI)	Specificity (95%-CI)	PPV (95%-CI)	NPV (95%-CI)
Screening approach	*N*	%	*N*				
1-visit^1^							
Screen-and-treat VIA	39	5.1	7	41 (18 - 67)	96 (94 - 97)	18 (8 - 34)	98.6 (97.5 – 99.3)
Screen-and-treat CareHPV	102	13.4	12	71 (44 - 90)	88 (85 - 90)	12 (6 - 20)	99.2 (98.2 – 99.8)
Screen-and-treat PCR	120	15.7	15	88 (64 - 99)	86 (83 - 88)	13 (7 - 20)	99.7 (98.9 - 100)
2-visit^2^							
Treatment based on careHPV	89	11.6	10	59 (33 - 82)	89 (87 - 92)	11 (6 - 20)	99.0 (97.9 – 99.6)
Treatment based on careHPV+VIA triage^3^	22	2.9	6	35 (14 - 62)	98 (97 - 99)	27 (11 - 50)	98.5 (97.4 – 99.3)
Treatment based on PCR	100	13.1	13	77 (50 - 93)	88 (86 - 91)	13 (7 - 21)	99.4 (98.5 – 99.8)
Treatment based on PCR + VIA triage	20	2.6	7	41 (18 - 67)	98 (97 - 99)	35 (15 - 59)	98.7 (97.5 – 99.4)

^1^ 3 hHSIL+ imputed among 29 women without a second visit are considered treated

^2^ 3 hHSIL+ imputed among 29 women without a second visit are considered untreated

^3^ Treatment based on careHPV and VIA triage was the screening approach used, according to Rwanda MoH screening recommendation [2]

*CI* confidence interval, *hHSIL* histological high-grade squamous intraepithelial lesions, *MoH* Ministry of Health, *NPV* negative predictive value, *PCR* polymerase chain reaction, *PPV* positive predictive value, VIA visual inspection with acetic acid

Table 2 also shows screening indices of cross-sectional accuracy based on a 2-visit approach, i.e., HPV primary screening followed by referral of all HPV-positive women for treatment, in which 29 women were lost to follow-up and would not be treated even if screen-positive. Treating all careHPV positive women in a 2-visit approach resulted in the treatment of 11.6% of women and was associated with a sensitivity of 59% against hHSIL+. Using VIA to triage careHPV-positives at the second visit resulted in the treatment of only 2.9% of women, but was associated with a drop in sensitivity to 35%.

A similar drop in sensitivity was observed for using VIA to triage PCR-positives in a 2-visit approach, from 73% for treatment of all PCR-positives, down to 47%. Of note, PCR also provided information on HPV genotype: 8 of the 11 PCR-positive hHSIL+ observed at the second visit were HPV16/18-positive (6 HPV16, 2 HPV18), of which 5 (62%) were VIA triaged to no treatment.

Relative performances of the different testing approaches were consistent when using cytological HSIL+ (cHSIL+) (Additional file 1: Table S2), or a composite cytological/histological HSIL+ endpoint (Additional file 1: Table S3), instead of hHSIL+. Furthermore, relative performances against hHSIL+ were also consistent in sensitivity analyses restricted to women aged 25-44 years only (Additional file 1: Table S4).

## Discussion

This evaluation of the performance of the 2013 Rwandan MoH cervical cancer screening campaign is the first real-world comparison of the three options endorsed by the 2013 WHO guidelines for screening and treatment of precancerous cervical lesions in LMICs. Although careHPV did not perform quite as well as a reference PCR HPV test, a careHPV screen-and-treat approach would have treated more hHSIL+ than VIA screen-and-treat (albeit with a higher treatment rate overall). In the actual 2-visit approach used in Rwanda, necessitated by the inability for rapid turnaround of careHPV results, an unacceptably high proportion of women with HPV-positive hHSIL+ were subsequently lost to follow-up and/or triaged by VIA to no treatment, with a result that the 2-visit approach of careHPV+VIA triage ended up performing similarly to VIA screen-and-treat.

In a 1-visit screen-and-treat approach, sensitivity of careHPV was substantially greater than that of VIA, as shown previously [19]. The 71% estimate of careHPV sensitivity represents that at the beginning of experience with the test platform in Rwanda, and was lower than that reported in previous large evaluations (84-100%) [20–24]. It was also lower than that of a HR-HPV PCR test (GP5+/6+) that was done on the same samples in a reference laboratory. Of note, careHPV was performed on clinician-collected samples in the current study, but has been shown to be similarly applicable to self-collected samples [21, 23, 25–27].

Although the initial plan of the Rwandan MoH was to deliver careHPV results on the same day [2] a 1-visit careHPV screen-and-treat approach proved unfeasible. This was partly due to the time requirement of collection of 90 samples coupled with a 3.5 h testing run, but was also hampered by a high number of invalid runs that required re-testing, which are technical features that are not expected to be shared by all HPV tests. Thus, careHPV-positive women were called back for triage and treatment in a second visit, which was associated with the loss to follow-up of an estimated 13% of careHPV-positive hHSIL+. Of note, the loss to follow-up in our well-supported research program is likely to be lower than in the wider Rwanda MoH campaign, and can be compared to 25-30% in other experiences of recalling HPV-positive women [6–8, 28].

The fact that not all careHPV-positive women were treated at the second visit but only those positive by a second VIA triage test, greatly reduced the number of women receiving treatment in Rwanda, from 13% to 3%. However, an important fraction (4 out of 10) careHPV-positive hHSIL+ seen at visit 2 were also triaged to no treatment, so that hHSIL+ sensitivity for careHPV+VIA triage dropped to 35%. These findings confirm those of similar studies in which a HPV + VIA triage algorithm was associated with sensitivity of only 33% for cervical intraepithelial neoplasia (CIN)2+ in Cameroon [7] and 34% for cHSIL+ in Papua New Guinea [29]. In other settings, the proportion of HPV-positive CIN2+ triaged to no treatment have been reported as 75% [6], 34% [8] or, estimated in the presence of verification bias due to lack of systematic biopsies, 18% [30].

Histologically confirmed HSIL is a more accurate diagnosis of true cervical precancerous lesions than cytological HSIL, and was considered as the gold standard outcome for screening indicators in our analysis. However, histology can also suffer from verification bias when biopsies are not obtained from all screened women. In the present study, we attempted to address this problem by obtaining histology from a high proportion of screen-positive women, by expert review of all biopsies according to recommended criteria [15], and by imputing underlying hHSIL+ among the few from whom we did not have biopsies. It is gold-standard to obtain biopsies directed by colposcopy, but in the present study, as in other similar evaluations [7], they were directed by VIA. Nevertheless, we were able to show that screening accuracy indices were consistent when evaluated against the outcome of cHSIL+ detected on liquid-based cytology samples that were collected from all women at the same visit as those for careHPV, and evaluated in The Netherlands blindly to HPV results.

We report on VIA as applied in a low-resource setting during the implementation of a government screening program, which is more likely to reflect real world conditions than those estimated from clinical trials, as VIA is subjective and known to vary in quality according to level of training of personnel. In addition to their previous local training, study health workers were nonetheless provided a refresher course on VIA and thermocoagulation prior to the start of the study. Despite this, the estimate of primary VIA screening sensitivity (41%) fell into the lower range of earlier estimates from similar studies [31], but comparable to that in other large real world experiences that also included widespread training and quality control [32, 33].

Our evaluation was associated with a number of weaknesses, most notably the small number of cases of hHSIL+, which affected the stability of estimates and prohibited the possibility of performing sub-analyses. For example, a substantial subset (35%) of our study population was HIV-positive. This should not explain the poor sensitivity of VIA, as positivity rate and sensitivity tends to be higher in HIV-positive persons for HPV and VIA alike [34]. Furthermore, our semi-observational study design prohibited the evaluation of whether VIA triage of careHPV positives in a 1-visit approach may have treated a few extra hHSIL+ that were subsequently lost to follow-up. Lastly, performance of the HPV + VIA triage algorithm might have improved if careHPV-positive VIA-negative women had been re-screened for persistent HPV-positivity at one year, according to the initial plan of the pilot screening campaign [2]. We were not able to assess this algorithm as, following the first round of screening, the Rwanda MoH decided not to continue with careHPV-testing.

## Conclusions

In summary, our data send a warning against the real-world utility of a 2-visit approach to VIA-triage of HPV-positive women as an algorithm for cervical cancer screening [6–8, 29]. Indeed, it appears that investment in HPV-testing to identify women at highest risk of hHSIL+ needs to be coupled with systematic treatment (where possible, in a single visit), to obtain the long term prevention of CIN2+ observed for HPV screen-and-treat approaches in prevention trials [35]. In this pragmatic “HPV screen-and-treat” approach, which has been put into practice in large implementation studies [25], VIA is still used, not to determine whether to treat or not, but to determine eligibility for cryotherapy/thermocoagulation. Although this approach will treat some women without disease, it will also destroy cells from the cervical squamocolumnar junction where most HPV-related neoplastic lesions originate [36]. Indeed, in a modelling exercise, even with re-screening at one year of HPV-positives, 2-visit VIA-triage of HPV positive women was not a cost effective approach in comparison to treating all HPV-positive women [37].

In the experience in Rwanda, the losses associated with requirement of a second visit and/or with VIA triage led to a 2-visit approach of careHPV+VIA triage treating a similar number of hHSIL (6 out of 17) as VIA screen-and-treat. Indeed, independently of the current analysis, the Rwanda MoH has decided, for pragmatic reasons, to resort to a recommendation of VIA screen-and-treat for cervical cancer screening for the time being.

## Additional file

Additional file 1: **Table S1.** Selected characteristics of the study population of 764 women aged 25-69 years. Rwanda 2013-14. **Table S2.** Sensitivity analysis: screening algorithm accuracy to detect 20 cytological (c) HSIL+ among 764 women aged 25-69 years. Rwanda 2013-14. **Table S3.** Sensitivity analysis: screening algorithm accuracy to detect 27 composite HSIL+ (cHSIL+ and/or hHSIL+) among 764 women aged 25-69 years. Rwanda 2013-14. **Table S4.** Sensitivity analysis: screening algorithm accuracy to detect 10 hHSIL+ among 428 women aged 25-44 years, after correction for missing data. Rwanda 2013-14 (DOCX 59 kb)

## Abbreviations

ASC-US: Atypical squamous cells of undetermined significance
cHSIL: Cytological high-grade squamous intraepithelial lesions
CI: Confidence interval
CIN: Cervical intraepithelial neoplasia
hHSIL: Histological high-grade squamous intraepithelial lesions
HR-HPV: High-risk human papillomavirus
IARC: International Agency for Research on Cancer
LMIC: Low- and middle-income country
LSIL: Low-grade squamous intraepithelial lesions
MoH: Ministry of Health
NPV: Negative predictive value
PCR: Polymerase chain reaction
PPV: Positive predictive value
VIA: Visual inspection with acetic acid
