# Supplementary material for: Evaluation of human-papillomavirus testing and visual inspection for cervical cancer screening in Rwanda
Source: BMC Womens Health. 2018 Apr 24;18:59. doi: 10.1186/s12905-018-0549-5 (PMC5921370; doi:10.1186/s12905-018-0549-5)
Supplement: Supplementary file 1 — Table S1. Selected characteristics of the study population of 764 women aged 25-69 years. Rwanda 2013-14. Table S2. Sensitivity analysis: screening algorithm accuracy to detect 20 cytological (c) HSIL+ among 764 women aged 25-69 years. Rwanda 2013-14. Table S3. Sensitivity analysis: screening algorithm accuracy to detect 27 composite HSIL+ (cHSIL+ and/or hHSIL+) among 764 women aged 25-69 years. Rwanda 2013-14. Table S4. Sensitivity analysis: screening algorithm accuracy to detect 10 hHSIL+ among 428 women aged 25-44 years, after correction for missing data. Rwanda 2013-14 (DOCX 59 kb) [file 12905_2018_549_MOESM1_ESM.docx]

**Additional file 1**

Provided by the authors to give readers additional information about their work.

**Additional file to:** Evaluation of human-papillomavirus testing and visual inspection for cervical cancer screening in Rwanda. Marie-Chantal Umulisa, Silvia Franceschi, Iacopo Baussano, Vanessa Tenet, Mathilde Uwimbabazi, Belson Rugwizangoga, Daniëlle A.M. Heideman, Anne M. Uyterlinde, Teresa M. Darragh, Peter J.F. Snijders, Felix Sayinzoga, Gary M. Clifford.

**Table S1.** Selected characteristics of the study population of 764 women

aged 25-69 years. Rwanda 2013-14

| **Characteristic** | ***N* women**^1^ | **%** |
| --- | --- | --- |
| **Total** | 764 | 100.0 |
|  |  |  |
| **Age-group (years)** |  |  |
| 25-34 | 154 | 20.1 |
| 35-44 | 274 | 25.9 |
| ≥45 | 336 | 44.0 |
|  |  |  |
| **HIV status** |  |  |
| Negative | 272 | 35.6 |
| Unknown | 225 | 29.4 |
| Positive | 267 | 35.0 |
|  |  |  |
| **Education (Years)** |  |  |
| Non-literate 0-5 | 121 | 15.8 |
| Literate 0-5 | 126 | 16.5 |
| Literate 6-10 | 350 | 45.8 |
| Literate ≥11 | 167 | 21.9 |
|  |  |  |
| **Marital status** |  |  |
| Married or cohabiting | 507 | 66.4 |
| Single | 54 | 7.0 |
| Separated/widowed | 203 | 26.6 |
|  |  |  |
| **Lifetime sexual partners** |  |  |
| 1 | 325 | 44.0 |
| 2 | 216 | 29.3 |
| 3 | 110 | 14.9 |
| ≥4 | 87 | 11.8 |
|  |  |  |
| **Previous cervical screening** |  |  |
| Yes | 20 | 2.6 |
| No | 737 | 97.4 |
|  |  |  |
| ^1^ Some figures do not add up the total due to missing values | | |

**Table S2.** Sensitivity analysis: screening algorithm accuracy to detect 20 cytological (c) HSIL+ among 764 women aged 25-69 years. Rwanda 2013-14

|  | **Women treated** | | **hHSIL+ treated** | **Sensitivity (95%-CI)** | **Specificity (95%-CI)** | **PPV**  **(95%-CI)** | **NPV**  **(95%-CI)** |
| --- | --- | --- | --- | --- | --- | --- | --- |
| **Screening approach** | ***N*** | **%** | ***N*** |  |  |  |  |
| **1-visit^1^** |  |  |  |  |  |  |  |
| Screen-and-treat VIA | 39 | 5.1 | 3 | 15 (3 - 38) | 95 (93 - 97) | 8 (2 - 21) | 97.7 (96.3 – 98.6) |
| Screen-and-treat CareHPV | 102 | 13.4 | 17 | 85 (62 - 97) | 89 (86 - 91) | 17 (10 - 25) | 99.5 (98.7 – 99.9) |
| Screen-and-treat PCR | 120 | 15.7 | 20 | 100 (83 - 100) | 87 (84 - 89) | 17 (11 - 25) | 100 (99.4 - 100) |
| **2-visit^2^** |  |  |  |  |  |  |  |
| Treatment based on careHPV | 89 | 11.6 | 14 | 70 (46 - 88) | 90 (88 - 92) | 16 (9 - 25) | 99.1 (98.1 – 99.7) |
| Treatment based on careHPV+VIA triage^3^ | 22 | 2.9 | 6 | 30 (12 - 54) | 98 (97 - 99) | 27 (11 - 50) | 98.1 (96.9 – 99.0) |
| Treatment based on PCR | 100 | 13.1 | 16 | 80 (56 - 94) | 89 (86 - 91) | 16 (9 - 25) | 99.4 (98.5 – 99.8) |
| Treatment based on PCR+VIA triage | 20 | 2.6 | 6 | 30 (12 - 54) | 98 (97 - 99) | 30 (12 - 54) | 98.1 (96.9 – 99.0) |
| ^1^ 4 cHSIL+ observed among 29 women without a second visit are considered treated. | | | | |  |  |  |
| ^2^ 4 cHSIL+ observed among 29 women without a second visit are considered lost to follow-up i.e. untreated.  ^3^ Treatment based on careHPV and VIA triage was the screening approach used, according to Rwanda MoH screening recommendation (Binagwaho et al, 2013).[1] | | | | | | |  |

cHSIL=cytological high-grade squamous intraepithelial lesions; CI=confidence interval; hHSIL=histological high-grade squamous intraepithelial lesions; HR-HPV=high-risk human papillomavirus; MoH=Ministry of Health; NPV=negative predictive value; PCR=polymerase chain reaction; PPV=positive predictive value; VIA=visual inspection with acetic acid.

**Table S3.** Sensitivity analysis: screening algorithm accuracy to detect 27 composite HSIL+ (cHSIL+ and/or hHSIL+) among 764 women aged 25-69 years. Rwanda 2013-14

|  | **Women treated** | | **hHSIL+ treated** | **Sensitivity (95%-CI)** | **Specificity (95%-CI)** | **PPV**  **(95%-CI)** | **NPV**  **(95%-CI)** | |
| --- | --- | --- | --- | --- | --- | --- | --- | --- |
| **Screening approach** | ***N*** | **%** | ***N*** |  |  |  |  |  |
| **1-visit^1^** |  |  |  |  |  |  |  | |
| Screen-and-treat VIA | 39 | 5.1 | 6 | 22 (9 - 42) | 96 (94 - 97) | 15 (6 - 31) | 97.1 (95.6 - 98.2) | |
| Screen-and-treat CareHPV | 102 | 13.4 | 22 | 82 (62 - 94) | 89 (87 - 91) | 22 (14 - 31) | 99.2 (98.2 - 99.8) | |
| Screen-and-treat PCR | 120 | 15.7 | 26 | 96 (81 - 100) | 87 (85 - 90) | 22 (15 - 30) | 99.8 (99.1 - 100.0) | |
| **2-visit^2^** |  |  |  |  |  |  |  | |
| Treatment based on careHPV | 89 | 11.6 | 19 | 70 (50 - 86) | 91 (88 - 93) | 21 (13 - 31) | 98.8 (97.7 - 99.5) | |
| Treatment based on careHPV+VIA triage^3^ | 22 | 2.9 | 9 | 33 (17 - 54) | 98 (97 - 99) | 41 (21 - 64) | 97.6 (96.2 - 98.6) | |
| Treatment based on PCR | 100 | 13.1 | 22 | 82 (62 - 94) | 89 (87 - 92) | 22 (14 - 31) | 99.2 (98.3 - 99.8) | |
| Treatment based on PCR+VIA triage | 20 | 2.6 | 10 | 37 (19 - 58) | 99 (98 - 99) | 50 (27 - 73) | 97.7 (96.4 - 98.7) | |
| ^1^ 3 composite HSIL+ observed among 29 women without a second visit are considered treated. | | | | | | | | |
| ^2^ 3 composite HSIL+ observed among 29 women without a second visit are considered lost to follow-up i.e. untreated.  ^3^ Treatment based on careHPV and VIA triage was the screening approach used, according to Rwanda MoH screening recommendation (Binagwaho et al, 2013).[1] | | | | | | | |  |

cHSIL=cytological high-grade squamous intraepithelial lesions; CI=confidence interval; hHSIL=histological high-grade squamous intraepithelial lesions; HR-HPV=high-risk human papillomavirus; MoH=Ministry of Health; NPV=negative predictive value; PCR=polymerase chain reaction; PPV=positive predictive value; VIA=visual inspection with acetic acid.

**Table S4.** Sensitivity analysis: screening algorithm accuracy to detect 10 hHSIL+ among 428 women aged 25-44 years, after correction for missing data. Rwanda 2013-14

|  | **Women treated** | | **hHSIL+ treated** | **Sensitivity (95%-CI)** | **Specificity (95%-CI)** | **PPV**  **(95%-CI)** | **NPV**  **(95%-CI)** | |
| --- | --- | --- | --- | --- | --- | --- | --- | --- |
| **Screening approach** | ***N*** | **%** | ***N*** |  |  |  |  |  |
| **1-visit^1^** |  |  |  |  |  |  |  | |
| Screen-and-treat VIA | 23 | 5.4 | 4 | 40 (12 - 74) | 96 (93 - 97) | 17 (5 - 39) | 98.5 (96.8 - 99.5) | |
| Screen-and-treat CareHPV | 61 | 14.3 | 6 | 60 (26 - 88) | 87 (83 - 90) | 10 (4 - 20) | 98.9 (97.2 - 99.7) | |
| Screen-and-treat PCR | 77 | 18.0 | 10 | 100 (69 - 100) | 84 (80 - 87) | 13 (6 - 23) | 100.0 (99.0 - 100.0) | |
| **2-visit^2^** |  |  |  |  |  |  |  | |
| Treatment based on careHPV | 52 | 12.1 | 5 | 50 (19 - 81) | 89 (85 - 92) | 10 (3 - 21) | 98.7 (96.9 - 99.6) | |
| Treatment based on careHPV+VIA triage^3^ | 13 | 3 | 4 | 40 (12 - 74) | 98 (96 - 99) | 31 (9 - 61) | 98.6 (96.9 - 99.5) | |
| Treatment based on PCR | 64 | 15 | 8 | 80 (44 - 98) | 87 (83 - 90) | 13 (6 - 23) | 99.5 (98.0 - 99.9) | |
| Treatment based on PCR+VIA triage | 12 | 2.8 | 5 | 50 (19 - 81) | 98 (97 - 99) | 42 (15 - 72) | 98.8 (97.2 - 99.6) | |
| ^1^ 3 composite HSIL+ observed among 16 women without a second visit are considered treated. | | | | | | | | |
| ^2^ 3 composite HSIL+ observed among 16 women without a second visit are considered lost to follow-up i.e. untreated.  ^3^ Treatment based on careHPV and VIA triage was the screening approach used, according to Rwanda MoH screening recommendation (Binagwaho et al, 2013).[1] | | | | | | | |  |

CI=confidence interval; hHSIL=histological high-grade squamous intraepithelial lesions; HR-HPV=high-risk human papillomavirus; MoH=Ministry of Health; NPV=negative predictive value; PCR=polymerase chain reaction; PPV=positive predictive value; VIA=visual inspection with acetic acid.

**Reference**

1. Binagwaho A, Ngabo F, Wagner CM, Mugeni C, Gatera M, Nutt CT, Nsanzimana S. Integration of comprehensive women's health programmes into health systems: cervical cancer prevention, care and control in Rwanda. Bull World Health Organ 2013;91:697-703.
